# Accessing Olfactory Habituation in *Drosophila melanogaster* with a T-maze Paradigm

**DOI:** 10.21769/BioProtoc.3259

**Published:** 2019-06-05

**Authors:** Ourania Semelidou, Summer F. Acevedo, Efthimios M.C. Skoulakis

**Affiliations:** 1 Division of Neuroscience, Institute of Basic Biomedical Research, Biomedical Sciences Research Centre “Alexander Fleming”, Vari, Greece; 2 School of Medicine, University of Crete, Heraklion, Greece

**Keywords:** Olfactory habituation, *D. melanogaster*, *Drosophila*, Habituation, Olfaction

## Abstract

Habituation is the process whereby perceptual changes alter the value of environmental stimuli, enabling salience filtering. This behavioral response decrement is a form of non-associative learning, where the subject learns about the stimulus and does not involve sensory adaptation, sensory or motor fatigue. The range of behavioral responses in *D. melanogaster* led to the development of a number of habituation paradigms addressing various sensory modalities. Habituation of osmotactic responses has previously been measured with the Y-maze test and required 30 min of odor exposure. Here, we describe an olfactory habituation assay utilizing the widely used in associative learning paradigms T-maze. Continuous or repetitive odor exposure for 4 min is adequate to attenuate osmotactic responses both to attractive and aversive odors. Importantly, the decreased response conforms to habitation parameters, presenting dishabituation and spontaneous recovery. This assay allows the study of habituation after brief odor exposure, but also discriminates between the two distinct phases of the response, an initial habituation latency period followed by habituation. In addition, the characterization of the neuronal circuits implicated in each phase facilitates further study of the molecular components underlying this process.

## Background

Habituation, the behavioral modification whereby responses to repeated inconsequential stimuli are attenuated, is highly conserved and has been studied in a wide range of species, from Aplysia to humans. In *Drosophila melanogaster*, various paradigms have been developed to study habituation to visual, mechanical, gustatory, and olfactory stimuli. The variety of fly responses to odor stimulation led to development of different habituation paradigms, including olfactory jump response habituation (Mihalek *et al.*, 1997; Asztalos *et al.*, 2007a and 2007b; Joiner *et al.*, 2007; Sharma *et al.*, 2009), olfactory startle response habituation (Cho *et al.*, 2004; Wolf *et al.*, 2007), unconditioned leg movement habituation (Chandra and Singh, 2005), and olfactory avoidance habituation (Das *et al.*, 2011).

Previous studies on olfactory avoidance habituation demonstrated that exposure to a continuous odor for 30 min attenuates the response, evidenced by subsequent testing in a Y-maze. Independently and in parallel with these studies, we developed an olfactory habituation paradigm using the T-maze, which is widely used in olfactory associative learning experiments and had already been in use for such experiments in the lab. Short repetitive or continuous odor exposure for a total of 4 min results in decreased responses both to aversive and to attractive odors. Importantly, this attenuated response complies with the parameters of habituation, as it recovers spontaneously and can be dishabituated (Thompson and Spencer, 1966; Rankin *et al.*, 2009). In addition, this paradigm facilitates the investigation of habituation latency, the initial process that precedes habituation, which may be linked to associative learning. In fact, because the equipment and mechanics of this olfactory habituation assay are similar to those used for classical odor discrimination-dependent associative learning, the paradigm is conducive to investigations of possible interdependence of these two processes. Lastly, this habituation paradigm has enabled identification of the neuronal subsets implicated in this process (Semelidou *et al.*, 2018), allowing the further study of the molecular pathways underlying habituation, in a neuronal-specific manner.

## Materials and Reagents

1. 14 ml Falcon tubes
2. Glass vials for odorants (diameter: 2.2 cm, height: 9.5 cm)
3. *Drosophila melanogaster*
4. 3-Octanol (CAS number: 589-98-0; ACROS Organics, catalog number: AC203770500), store at RT
5. Benzaldehyde (CAS number: 100-52-7; Sigma, catalog number: 418099), store at 4 °C
6. Ethyl acetate (CAS number: 141-78-6; Sigma, catalog number: 34858), store at RT
7. Butanedione (CAS number: 431-03-8; Sigma, catalog number: B85307), store at RT
8. Brewers Yeast (CAS number: 68876-77-7; ACROS Organics, catalog number: AC368080010), store at 4 °C
9. *Drosophila* food (see Recipes)
  - Semolina
  - Whole wheat flour
  - Brown sugar
  - Fructose
  - Soy flour
  - CaCl_2_
  - Dry yeast
  - Nipagen
  - Propionic acid

## Equipment

1. T-maze (Figures 1-3, Video 1):
  - One Plexiglass side panel with single and another of equal size but with two openings (height: 15 cm, width: 3.8 cm, thickness: 1.8 cm).
  - One Plexiglass elevator panel with a single opening and a “training point” (height: 15 cm, width: 3.8 cm, thickness: 1.8 cm). All openings have 1.8 cm diameter and are fitted on the inner side of the side panels that come in contact with the elevator, with Teflon O-rings. The measurements between the openings on the sides and the elevator are depicted in Figure 1. The elevator “training point” (height: 1.3 cm) (Figures 1 and 3) consists of smaller openings in a configuration 3-7-9-9-10-10-10-9-9-7-3, both in width and height. Vacuum ports on the back of the elevator have 1 cm diameter. They are fitted with a plastic or teflon barbed vacuum port adaptor (length: 3.7 cm) and a piece of silicon rubber tubing (diameter 0.6 cm ID Small Parts inc # B-210015). If additional fine control of incoming vacuum is required, then the silicon tubing can be cut and the two parts connected together with polycarbonate/polyethylene one-way stopcocks (Small Parts Inc # B-LSCP-100C).
  - One Plexiglass base (length: 10.5 cm, width: 5.2 cm, height: 1.8 cm) with four screw receptacles (upper diameter 1 cm, bottom diameter 0.6 cm, height: 1.6 cm) to receive aluminum screws as appropriate. The distance between two individual receptacles is 2.1 cm in length and 1.6 cm in width.
  - Aluminum hex head cap screws (length with head: 2.4 cm), silicon tube (diameter: 1 cm), “male” part of the pair of Nalgene Quick Disconnect to connect to home vacuum, O-rings (2.2 cm diameter and 2.0 mm thick).
  **Figure 1.**
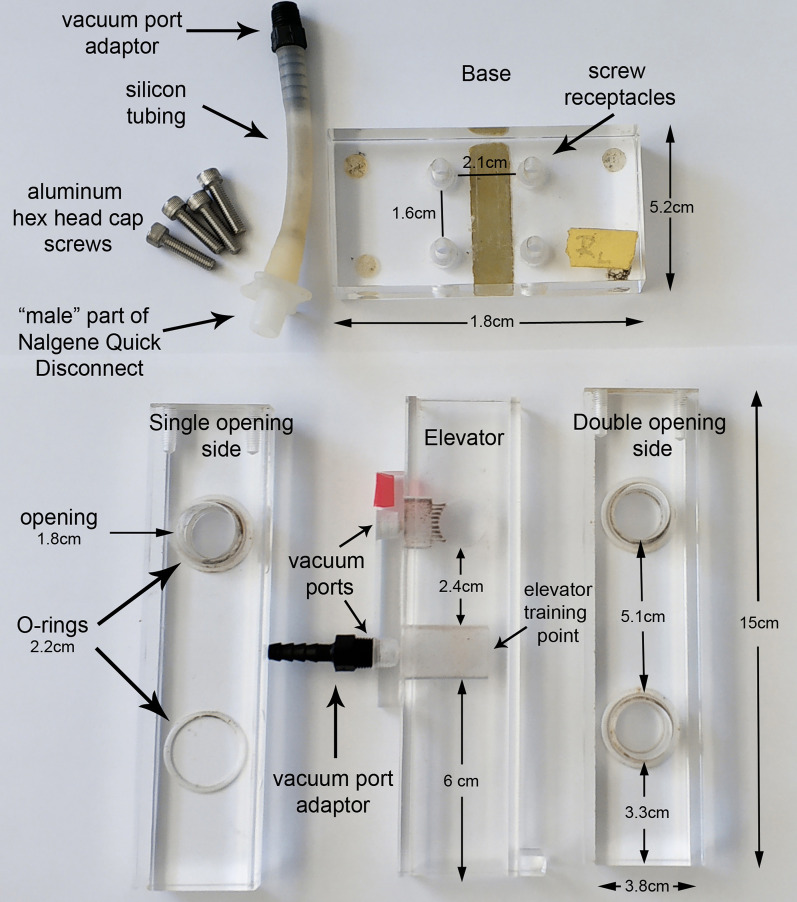
Depiction of the T-maze parts
  **Figure 2.**
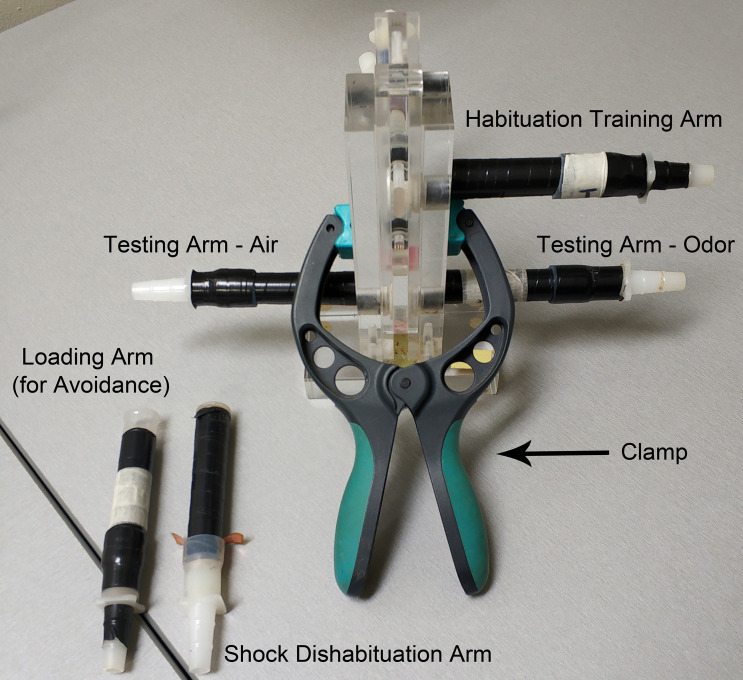
Olfactory habituation T-maze with fitted training and testing arms
  **Figure 3.**
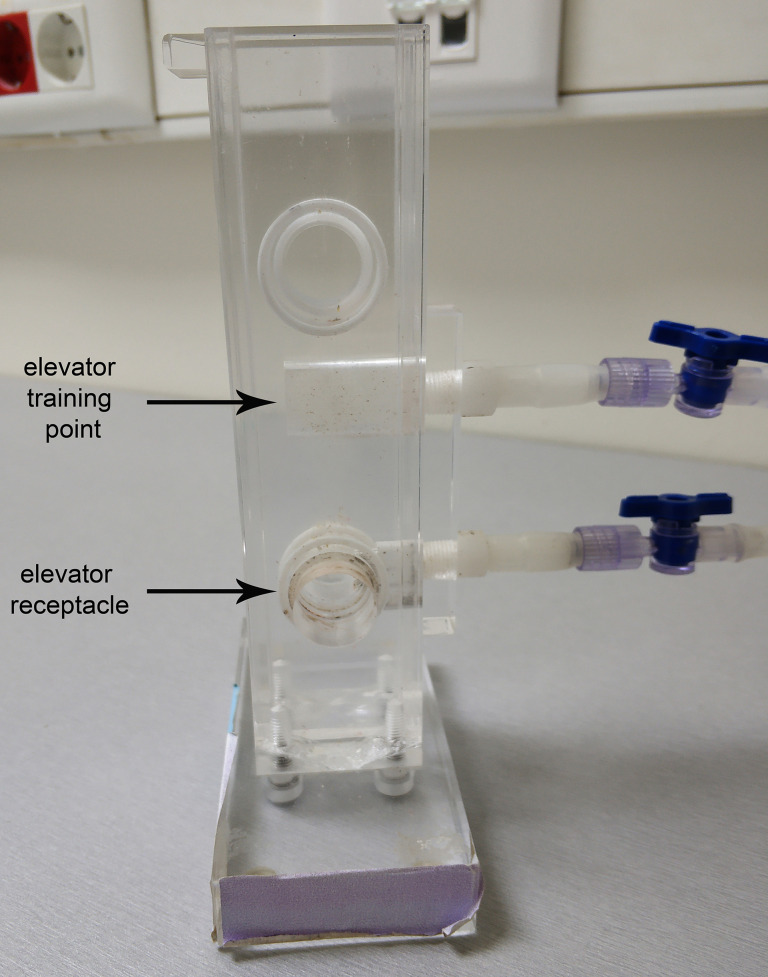
Side view of the T-maze. The training point and receptacle of the elevator are indicated.
2. 14 ml polypropylene Round bottom tubes (Falcon, catalog number: 352006)
3. Custom-made T-maze arms using Falcon 352006 tubes cut at the 1 ml mark (Figures 2 and 6)
These are fit at the now open “bottom” end with the “female” part of the pair of Nalgene Quick Disconnect, HDPE size 3/8 to 7/16 inch, which has been fitted on its wide end with monofilament cloth Nylon Mesh (1 mm opening, Small Parts Inc. #B-CMN-1000 or -500) (Figures 4 and 6) and attached to the Falcon tube with a piece 3-3.5 cm in length silicon rubber tubing (diameter: 1.4 cm ID, Small Parts Inc .# B-210025) (Figure 4)
4. Glass vials (diameter: 2.2 cm, height: 9.5 cm) (Figure 5)
5. Odor vial caps: Two-hole rubber stoppers #2 for the 14 ml tubes (Top: 20 mm, Bottom: 16 mm, Length: 25 mm) and #4 for the glass vials (Top: 25 mm, Bottom: 20 mm, Length: 25 mm), penetrated with two 0.3 cm glass tubes (diameter: 3 mm, length 5.5 cm for the long and 2 cm for the short). Silicon tubing (diameter: 4 mm Small Parts Inc # B-210010) to cover the upper part of the glass tubes and a “male” and “female” part of the pair of Nalgene Quick Disconnect, HDPE size 3/8 to 7/16 inch (Figure 5)
6. Custom made Copper grids (width: 5.9 cm, length: 8.5 cm) (Figure 6)
7. Adjustable house vacuum
8. Gilmont flowmeter (Thermo Scientific, catalog number: GF-2200)
9. Astro-Med/Grass technologies S48 Square Purse Stimulator
*
Note: Different versions of T-mazes can be found and purchased from http://www.celexplorer.com/.
*
  **Figure 4.**
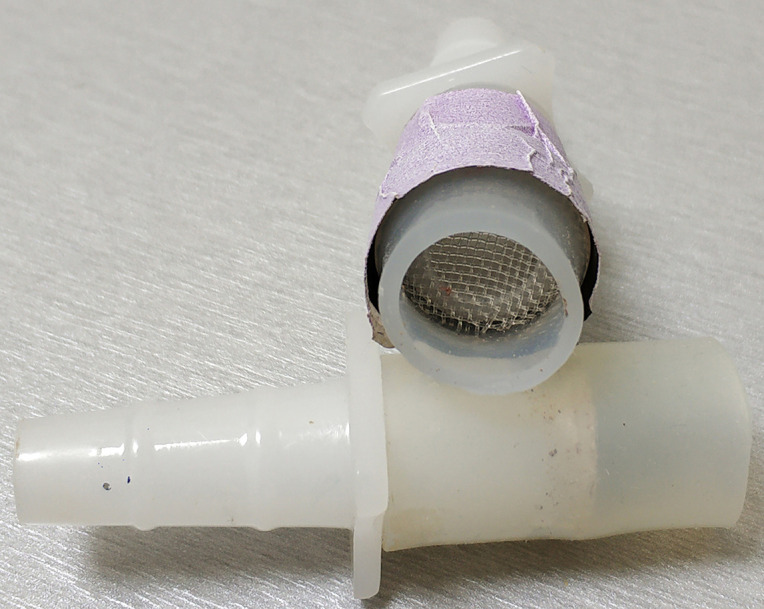
The “female” part of the pair of Nalgene Quick Disconnect, fitted on its wide end with monofilament cloth Nylon Mesh (1 mm opening). The silicon tubing is used to attach it to the Falcon tubes used as arms.
  **Figure 5.**
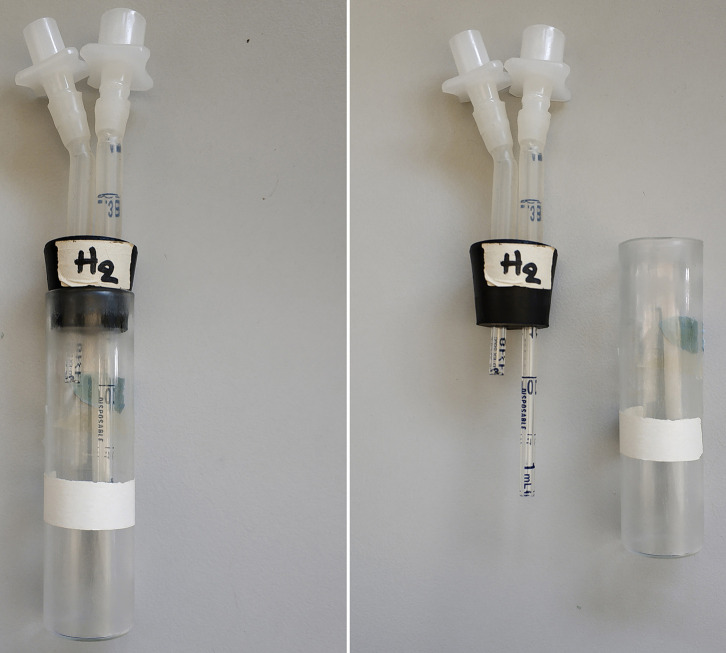
Odor vial cap made with rubber stopper #4 fitted into the odor glass vial, and the two parts demonstrated separately
  **Figure 6.**
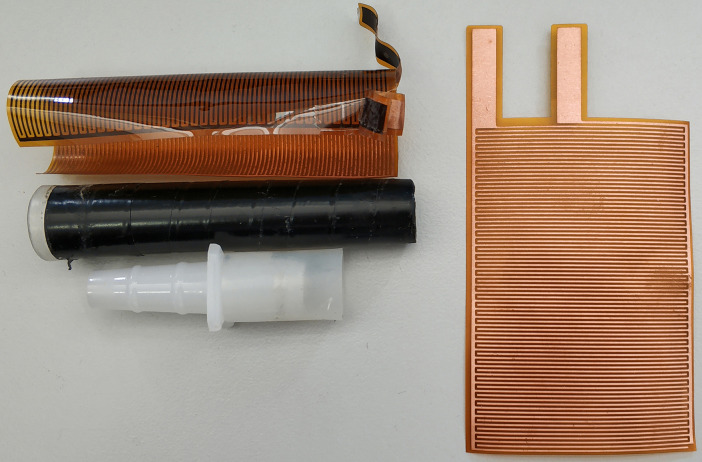
Shock dishabituation arm. The shock dishabituation arm consists of a copper grid, a cut Falcon tube and the “female” part of the pair of Nalgene Quick Disconnect with the silicon tubing attached. A new copper grid is demonstrated on the right.

## Software

1. JMP (by SAS, https://www.jmp.com/en_us/home.html), or any other statistics software

## Procedure

- T-maze assembly (Video 1)
  **Video 1.**
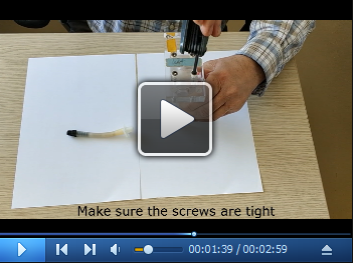
Demonstration of the T-maze construction
  The plexiglass components of the maze are assembled by first attaching the side panels and the elevator to the base with the aluminum screws. When all four screws are in place, tighten them crosswise to the point that they are tight without forcing them further. If forced too tight it may damage the plexiglass screw threads. Once the sides are fastened on the base, attach the vacuum adaptors onto the vacuum ports. Make sure that the elevator is snug, but it can move up and down without difficulty.
- Fly preparation
  1. Backcross control flies carrying the *w1118* mutation to the Canton-S for at least 10 generations to obtain the Cantonised-*w1118.* Outcross all the *Drosophila* lines with Cantonised-*w1118* for six generations to obtain the same genetic background for all animals used in the behavioral experiments.
  2. Raise the flies in standard wheat-flour-sugar food supplemented with soy flour and CaCl_2_ under a 14:10 h light-dark cycle, 60% relative humidity, at 25 °C, unless you use the TARGET system. In that case, raise the flies at 18 °C until hatching.
  3. To collect flies for the experiment, anesthetize them under CO_2_ at least one day before the experiment and separate them in groups of 50-60 flies. Place each group of flies in food vials at 25 °C, 14:10 h light-dark conditions and 60% humidity, if no transgene will be expressed during the experiment (for control experiments or mutants). For experiments with Gal4 lines, place the flies at 30 °C overnight to enhance transgene expression. For experiments using the TARGET system, place the flies at 30 °C for 2 or 3 days prior to testing. The days of transgene induction depend on the transgene used in each experiment. For neuronal silencing experiments with Shibire^ts^, place the flies at 32-33 °C for 30 min before the experiment, while for experiments with TRPA1 for neuronal activation, transfer the flies at 30-31 °C for the time period you want to keep the neurons activated.
- Preparation before the experiment
  1. Transfer the flies in new food vials approximately 1 h before the experiment. Place the vials in a dark box and keep it at the temperature flies were kept before. For experiments with Shibire^ts^, transfer the flies in new pre-warmed vials, kept at 32-33 °C, 30 min before the experiment. To ensure that neuronal transmission is blocked for the same time interval for all groups of flies, transfer the flies at 32-33 °C sequentially during the experiment.
  2. Prepare the behavior room. Clean the T-mazes and arms with soft cloth. Ensure that the arms fit tightly on the maze and the air flow is stable at 500 ppm (0.5 ml/min). If you use the T-maze for the first time, run a control experiment without odors to verify that there is no bias towards one arm of the maze. Check that humidity in the room ranges from 60% to 70% and the temperature from 23 to 24 °C. Experiments are performed under dim red light (photography dark room grade, Figure 7).
  3. Prepare the odor and connect it to the T-maze set up. Let the odor flow for 30 min, to prime the system for the experiment. For Octanol add 1 ml 3-Octanol in a glass vial. For experiments conducted with Benzaldehyde, add 100 μl of Benzaldehyde in a 14 ml Falcon. Similarly, for experiments with ethyl acetate or 2,3-butanedione prepare priming in a 14 ml Falcon with 10 μl of a 0.1% dilution of ethyl acetate in water and for 2,3-butanedione with 10 μl of a 0.5% dilution. The concentration of each compound and the surface area of the container (odorant meniscus) were determined empirically to produce the optimal response. Previous studies have demonstrated that the same odor compound can be either aversive or attractive, depending on the concentration employed (Wang *et al.*, 2003). For odorants different than the ones described herein, avoidance experiments are required prior to habituation to standardize the odor concentrations. These odorants can be diluted either in water (ethyl acetate and 2,3-butanedione) or isopropyl myristate (Octanol, Benzaldehyde [Gouzi *et al.*, 2018])
    **Figure 7.**
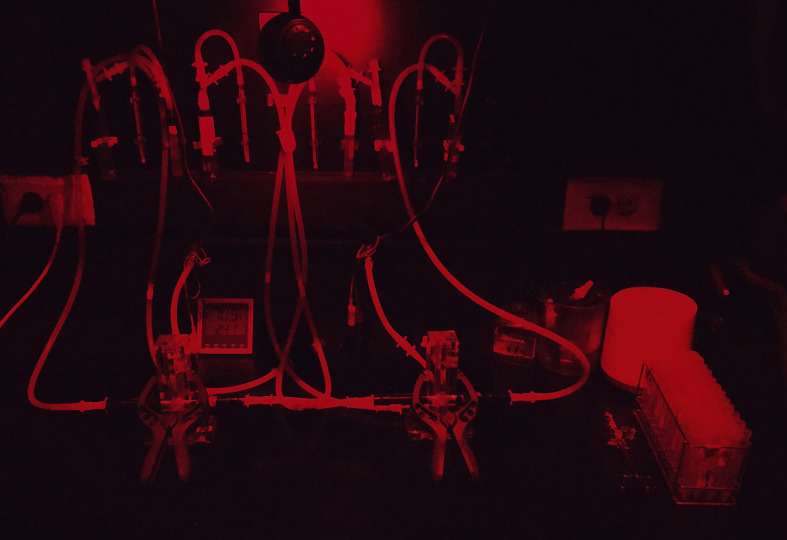
The equipment set under the conditions of the experiment. All experiments must be conducted under dim red light.
- Odor Avoidance and Attraction Testing
  1. Use a clamp to hold the parts of the T-maze together tightly (Figure 2). Load the flies in a clean arm and connect it on the upper part of the maze. Move the middle part of the maze (the elevator) so that the elevator receptacle (Figure 3) will be aligned with the arm. Tap the maze gently to transfer the flies into the receptacle and slide the elevator down quickly to trap the flies inside the maze.
  2. Connect the air and odor tubing, so that air will flow on one side of the maze and odor on the other.
  3. Connect the vacuum.
  4. Slide the elevator down and let the flies choose between the two arms for 90 s for aversive odors and 180 s for attractive odors. Make sure that during testing the flow remains at 500 ppm.
  5. Move the elevator up to trap the flies inside the two arms.
  6. Transfer the content of each arm to separate (numbered) tubes.
  7. Clean the elevator receptacle to remove any remaining flies, attach the arms back on the maze and proceed with the next n.
  8. Repeat the procedure until you finish with all the repetitions of the experiment.
  9. Transfer the rack where you have collected the flies to -80 °C. Wait for approximately 15 min and then count the flies. Note the number of flies trapped in the air-arm and the odor-arm, as well as their genotype.
- Habituation Training and Testing (Video 2)
  **Video 2.**
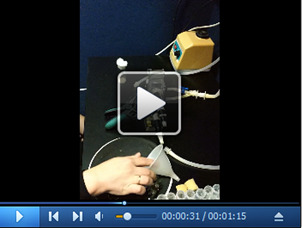
Olfactory Habituation Training and Testing
  1. Load the flies in an arm used specifically for training exposure to a particular odor and connect the arm on the upper part of the maze. Make sure you use different arms for each odor used in the assay if more than one odor is to be used.
  2. Slide the elevator so that the flies will stay trapped in the arm (elevator training point, Figure 3), but the odor can flow through it.
  3. Connect the vacuum tubing and then the odor tubing. Make sure that the odor flow is at 500 ppm.
  4. Leave the flies in the arm with the odor flow for the designated amount of time–1 min for habituation latency experiments, and 4 min or 30 min for habituation experiments. For odor pulse experiments substitute 1 min odor exposure with 2 x 30 s (with 8 s interstimulus interval), 4 min with 4 x 1 min (with 15 s interstimulus interval), and 30 min with 3 x 10 min (with 2.5 min interstimulus interval) odor pulses.
  5. Disconnect the odor tubing but leave the vacuum tubing connected such that air will flow through the arm. Wait for 30 s.
  6. Slide the elevator so that the receptacle will be aligned with the training arm. Tap the maze gently to transfer the flies in the receptacle and slide the elevator down quickly to trap the flies inside the maze (Video 2).
  7. Continue the procedure from “Step C2”.
- Dishabituation with electric shock
  1. Set the Grass Stimulator to 1.2 s stimulus duration at 45 V.
  2. Load the flies in an arm with a custom-made copper grid (Figure 6) and connect the arm on the upper part of the maze.
  3. Train the flies with 1, 4, or 30-min odor exposure as above. During this interval, connect the crocodile clips holding the electric shock wires with the extending parts of the copper grid.
  4. Disconnect the odor tubing and immediately apply one electric shock.
  5. Disconnect the crocodile clips and wait for 30 s with the vacuum on, so that air will flow through the arm.
  6. Slide the elevator so that the receptacle will be aligned with the copper-grid arm. Tap the maze gently to transfer the flies in the hollow of the elevator. Slide the elevator down quickly to trap the flies inside the maze.
  7. Continue the procedure from “Step C2”.
Previous studies have shown that concurrent exposure to an odor and twelve 45 V electric shocks results in associative learning formation (Berry *et al.*, 2018). Dishabituation, however, requires stimulation with a single electric shock following the odor exposure and therefore no associative learning formation is anticipated from the application of the protocol.
- Dishabituation with vortex
  1. Train the flies with 1, 4, or 30-min odor exposure as above.
  2. Disconnect the odor tubing and remove the arm from the maze, sealing it with your hand. Apply vortex for 3 s at maximum speed.
  3. Connect the arm on the maze again and connect the vacuum tubing so that air will flow through the arm. Wait for 30 s.
  4. Slide the elevator so that the receptacle will be aligned with the training arm. Tap the maze gently to transfer the flies in the elevator receptacle and slide the elevator down quickly to trap the flies inside the maze.
  5. Continue the procedure from “Step C2”.
- Dishabituation with yeast puff
  1. Prepare a 30% solution of yeast in water, an arm and tubing that will be used specifically for this odor. Make sure that the odor flows with 500 ppm.
  2. Train the flies with 1, 4, or 30-min odor exposure as above.
  3. Disconnect the odor tubing and remove the arm from the maze, sealing it with your hand. Transfer the flies to the new arm, specifically used for yeast puff dishabituation. Connect the tubing for yeast puff for 3 s.
  4. Remove the yeast puff tubing and leave the vacuum tubing on so that air will flow through the arm. Wait for 30 s.
  5. Slide the elevator so that the receptacle will be aligned with the training arm. Tap the maze gently to transfer the flies in the receptacle and slide the elevator down quickly to trap the flies inside the maze.
  6. Continue the procedure from “Step C2”.
- Spontaneous recovery
  Proceed as described in ‘Procedure D: Habituation training and testing’. After Step D5, transfer the flies to food vials for 6 min. Continue with Step D6.

## Data analysis

1. Open JMP and create a new data table.
2. Name the columns as Genotype-Air-[Odor name]–PI [Odor] *100 (Figure 8).
  **Figure 8.**
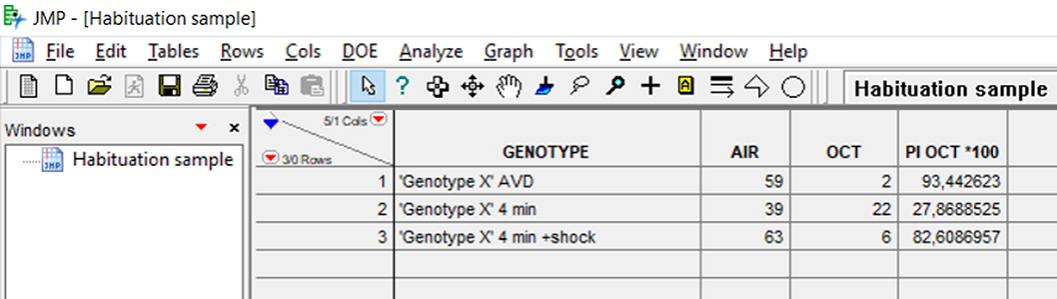
JMP data table for Habituation to OCT and Dishabituation with shock
3. Add formulas for ‘PI [Odor] *100’. To add a formula on a column, right click on the name of the column and choose ‘Formula’. The formula for ‘PI [Odor] *100’ is: [(AIR - OCT)/(AIR + OCT)] * 100.
4. Add the genotypes and the type of training (Avoidance, [minutes of odor] for habituation training, [minutes of odor + shock] for dishabituation training with electric shock, *etc*.).
5. Transfer your results in the data table. ‘Air’ indicates the number of flies that chose the arm with air, while [Odor] indicates the number of flies that chose the arm with the odor.
6. When you have completed enough experiments and have approximately 10 ns/genotype for each treatment you can proceed with the statistical analysis.
7. For the statistical analysis concatenate the JMP files from all experiments and create a ‘Fit X by Y’ distribution by the ‘Analyze’ tool. Add ‘Genotype’ as the X, Factor and PI [Odor] *100 as the Y, Response (Figure 9). In the graphic representation choose ‘Means/Anova’ from the top left red arrow. In case ANOVA shows *P* < 0.01, proceed with further analysis. For Dunnett’s test, choose ‘Compare means’ from the top left red arrow and then ‘With Control, Dunnett’s’. Dunnett’s must be applied for each genotype individually.
  **Figure 9.**
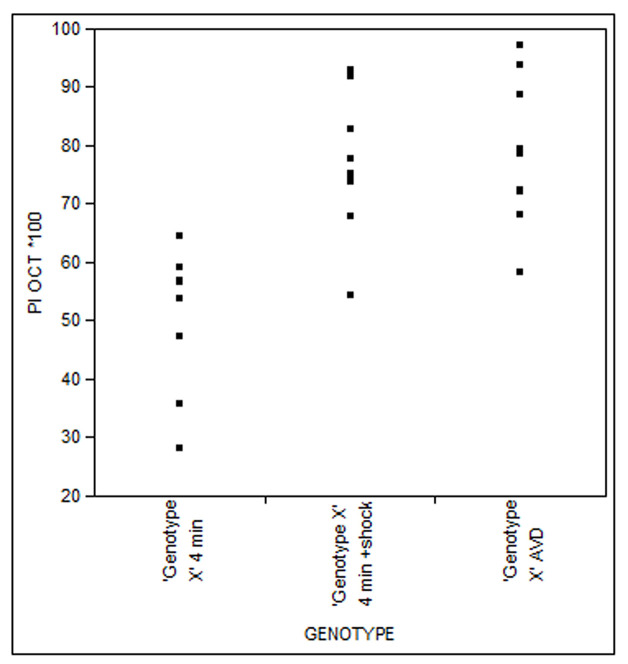
Example of Fit Y by X analysis of a 4 m Habituation experiment and Dishabituation with electric shock

## Notes

Perform experiments by random, pseudorandom or blind but balanced design of ns for Avoidance, Habituation and Dishabituation testing.

## Recipes

1. *Drosophila* food
  140 g semolina
  180 g whole wheat flour
  180 g brown sugar
  72 g fructose
  15 g soy flour
6 g CaCl_2_
  210 g dry yeast
  150 ml Nipagen (10% in EtOH)
  25 ml Propionic acid
  Bring up to 8.5 L final volume with house distilled water
